# Improved optical performance of multi-layer MoS_2_ phototransistor with see-through metal electrode

**DOI:** 10.1186/s40580-019-0202-5

**Published:** 2019-10-02

**Authors:** Junghak Park, Dipjyoti Das, Minho Ahn, Sungho Park, Jihyun Hur, Sanghun Jeon

**Affiliations:** 1 Department of Applied Physics, Korea University, 2511 Sejong-ro, Sejong city, 30019 Republic of Korea; 2 School of Electrical Engineering, Korea Advanced Institute of Science and Technology, Yuseong, Daeharkro 291, Dajeon-city, Republic of Korea; 3 Division of Life Science and Chemistry, Daejin University, 1007, Hguk-ro, Pochehon city, Gyeonggi-do 487-711 Republic of Korea; 4 Department of Electrical Engineering, Sejong University, 209, Neungdong-ro, Gwangjin-gu, Seoul-city, 05006 Republic of Korea

**Keywords:** Barrier height, MoS_2_, Phototransistor, Photocurrent, See-through metal electrode

## Abstract

In recent years, MoS_2_ has emerged as a prime material for photodetector as well as phototransistor applications. Usually, the higher density of state and relatively narrow bandgap of multi-layer MoS_2_ give it an edge over monolayer MoS_2_ for phototransistor applications. However, MoS_2_ demonstrates thickness-dependent energy bandgap properties, with multi-layer MoS_2_ having indirect bandgap characteristics and therefore possess inferior optical properties. Herein, we investigate the electrical as well as optical properties of single-layer and multi-layer MoS_2_-based phototransistors and demonstrate improved optical properties of multi-layer MoS_2_ phototransistor through the use of see-through metal electrode instead of the traditional global bottom gate or patterned local bottom gate structures. The see-through metal electrode utilized in this study shows transmittance of more than 70% under 532 nm visible light, thereby allowing the incident light to reach the entire active area below the source and drain electrodes. The effect of contact electrodes on the MoS_2_ phototransistors was investigated further by comparing the proposed electrode with conventional opaque electrodes and transparent IZO electrodes. A position-dependent photocurrent measurement was also carried out by locally illuminating the MoS_2_ channel at different positions in order to gain better insight into the behavior of the photocurrent mechanism of the multi-layer MoS_2_ phototransistor with the transparent metal. It was observed that more electrons are injected from the source when the beam is placed on the source side due to the reduced barrier height, giving rise to a significant enhancement of the photocurrent.

## Introduction

Molybdenum disulfide (MoS_2_), a typical transition metal dichalcogenide (TMDC) material, is attracting significant attention from researchers in the field of future optoelectronic devices due to its excellent optical as well as electrical properties, such as a high absorption coefficient, narrow bandgap, and high carrier mobility [1–5]. In recent years, MoS_2_ has been studied extensively in relation to thin film transistor (TFT) technology, and TFTs composed of multi-layer MoS_2_ have been found to exhibit useful features of future switching devices, such as large on/off current ratios, high field effect mobility values (μ_FE_), low temperature processes, and low power consumption [6, 7]. Especially due to its narrow bandgap, MoS_2_ has emerged as a prime material for photodetector as well as phototransistor applications, demonstrating the potential to outperform graphene by demonstrating better light responsiveness [8–12]. MoS_2_, however, exhibits a direct or an indirect energy bandgap property based on the number of layers [13]; therefore, its carrier mobility, absorbance and luminescence properties as well as its structural properties all strongly depend on the number of layers [14–16]. Multi-layer MoS_2_ has a higher density of state and a relatively narrow bandgap as compared to mono-layer MoS_2_ which can be advantageous for phototransistor applications [17]. However, unlike mono-layer MoS_2_, the multi-layer MoS_2_ has an indirect bandgap characteristic and therefore multi-layer MoS_2_ phototransistors possess inferior optical properties as compared to mono-layer MoS_2_ phototransistors [9].

The optical properties of the multi-layer MoS_2_ phototransistors can be improved significantly by utilizing transparent electrodes. However, the high work function of conventional transparent electrodes often gives rise to a large Schottky barrier and limits device performance [18]. The proper choice of transparent electrode is therefore of utmost importance to achieve high performance from MoS_2_ phototransistors. In this study, we investigate the electrical as well as the optical properties of single-layer as well as multi-layer MoS_2_-based phototransistors and demonstrate improved optical properties of multi-layer MoS_2_ phototransistors through the use of see-through metal electrodes instead of traditional global bottom gate or patterned local bottom gate structures. An increase in the dark-state ON current as well as the photocurrent in an illuminated state was observed when increasing the MoS_2_ thickness from the monolayer to the bulk due to the increase in the carrier concentration along with an increase in the decay time, as revealed by persistent photoconductivity (PPC) measurements. The see-through metal electrode utilized in this study was found to exhibit transmittance of more than 70% under visible light at 532 nm, thereby allowing the incident light to reach the entire active area below the source and drain electrodes. To investigate the effect of the contact electrodes on MoS_2_ phototransistors further, phototransistors with conventional opaque electrodes and transparent IZO electrodes were fabricated and compared to the proposed electrode. To gain better insight into the behavior of the photocurrent mechanism of the multi-layer MoS_2_ phototransistor with the see-through metal electrode, position-dependent photocurrent measurements were also carried out by locally illuminating the MoS_2_ channel at different positions.

## Experiment

The MoS_2_ phototransistors were fabricated in a conventional inverted staggered gate structure. Each MoS_2_ flake was mechanically exfoliated from a bulk MoS_2_ crystal and transferred to the top of a highly doped p-type Si wafer with a SiO_2_ thickness of 3000 Å. Highly doped p++ silicon and silicon dioxide layers were used as the back gate and gate insulator, respectively. After transferring the MoS_2_ onto the substrate, source and drain electrodes were patterned by conventional photolithography. Finally, the Ti/Au metal was deposited by electron beam evaporation as a contact electrode. The formation of single-layer as well as multi-layer MoS_2_ was confirmed with the help of AFM and Raman spectroscopy. The electrical and the optical properties of the TFTs were measured using a semiconductor device analyzer. A monochromator with wavelengths within the visible light region (400–900 nm) was employed to measure the optical characteristics of the individual phototransistors. In particular, a beam with a wavelength of 532 nm and with a radius of 1 µm in the Raman spectroscope was utilized to carry out the position-dependent photocurrent measurements of the TFTs.

## Results and discussion

**Fig. 1:**
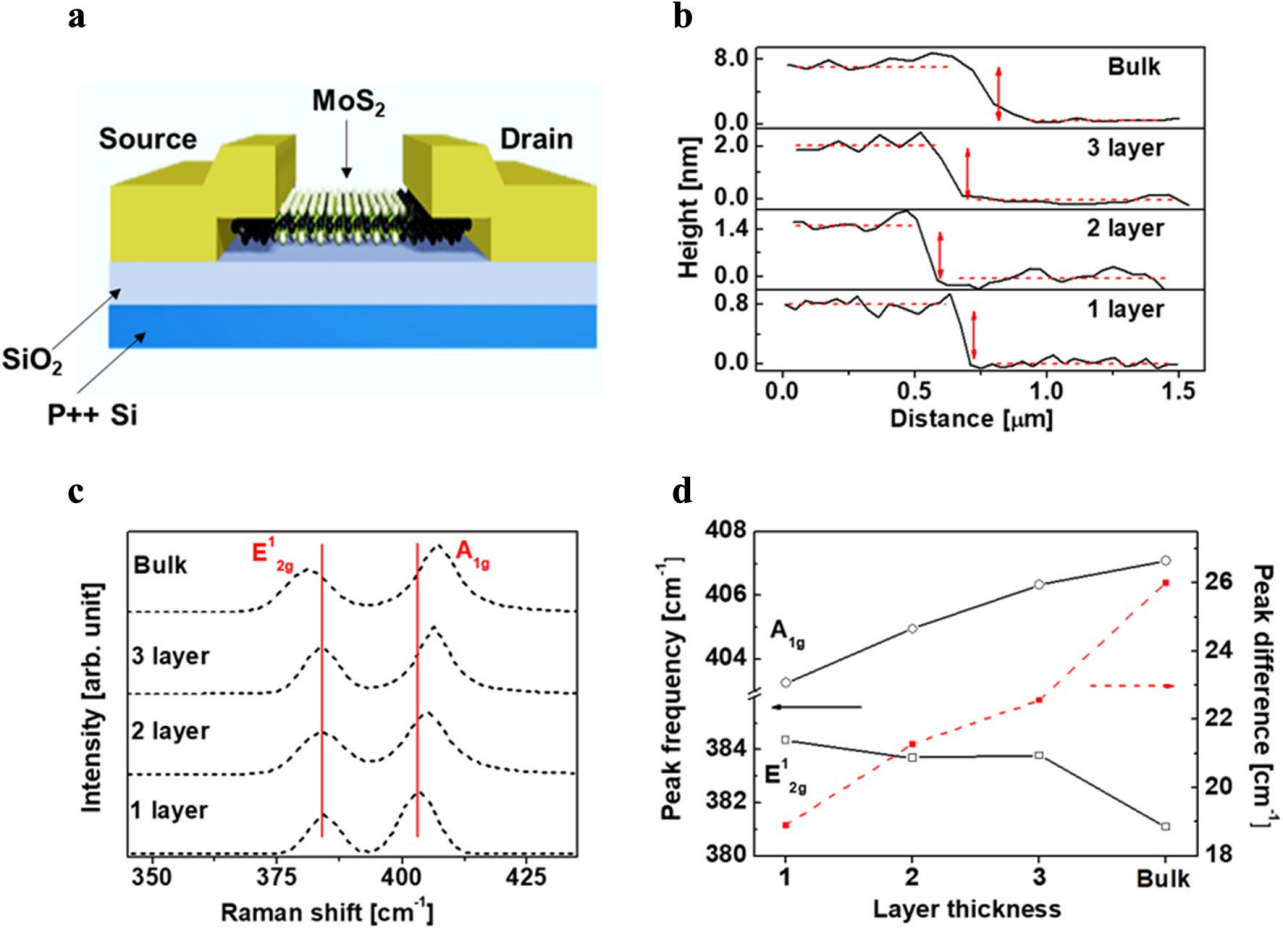
**a** Schematic diagram of the conventional bottom gate structure MoS_2_ photo-TFT, **b** height profile of the MoS_2_ layers, **c** Raman spectra of MoS_2_ samples with different numbers of layers. The left and right red lines indicate the positions of the ${\text{E}}^{ 1}_{{ 2 {\text{g}}}}$ and A_1g_ peaks in the mono-layer MoS_2_, respectively. **d** Frequencies of the ${\text{E}}^{ 1}_{{ 2 {\text{g}}}}$ and A_1g_ Raman peaks (left side axis) and their difference (right side axis) with different numbers of MoS_2_ layers

Figure 1a shows the schematic diagram of the fabricated MoS_2_ photo-TFT with the conventional bottom gate structure. The number of MoS_2_ layers in the as-fabricated phototransistor was confirmed from the AFM height profiles, as depicted in Fig. 1b. As shown in the figure, the height of the mono-layer MoS_2_ on the substrate is around 0.7 nm or more, which is slightly higher than the theoretical thickness of 6.15 Å due to the absorber on the MoS_2_ surface. Figure 1c presents the Raman spectrum of MoS_2_ at different thicknesses. The ${\text{E}}^{ 1}_{{ 2 {\text{g}}}}$ mode close to 383 cm^−1^ and the A_1g_ mode close to 408 cm^−1^ are observed from the mono-layer to the bulk MoS_2_. As shown in Fig. 1d, with an increase in the number of MoS_2_ layers, the frequency of the ${\text{E}}^{ 1}_{{ 2 {\text{g}}}}$ peak decreases whereas that of the A_1g_ peak increases. An increase in the number of MoS_2_ layers resulted in a decrease in the Van der Waals force [19] between adjacent layers, causing a red shift of the ${\text{E}}^{ 1}_{{ 2 {\text{g}}}}$ peak. Moreover, the Van der Waals force at each MoS_2_ layer suppresses the vibration as the number of layers is increased. This produces a higher force constant [20], resulting in a blue shift of the A_1g_ modes.

**Fig. 2:**
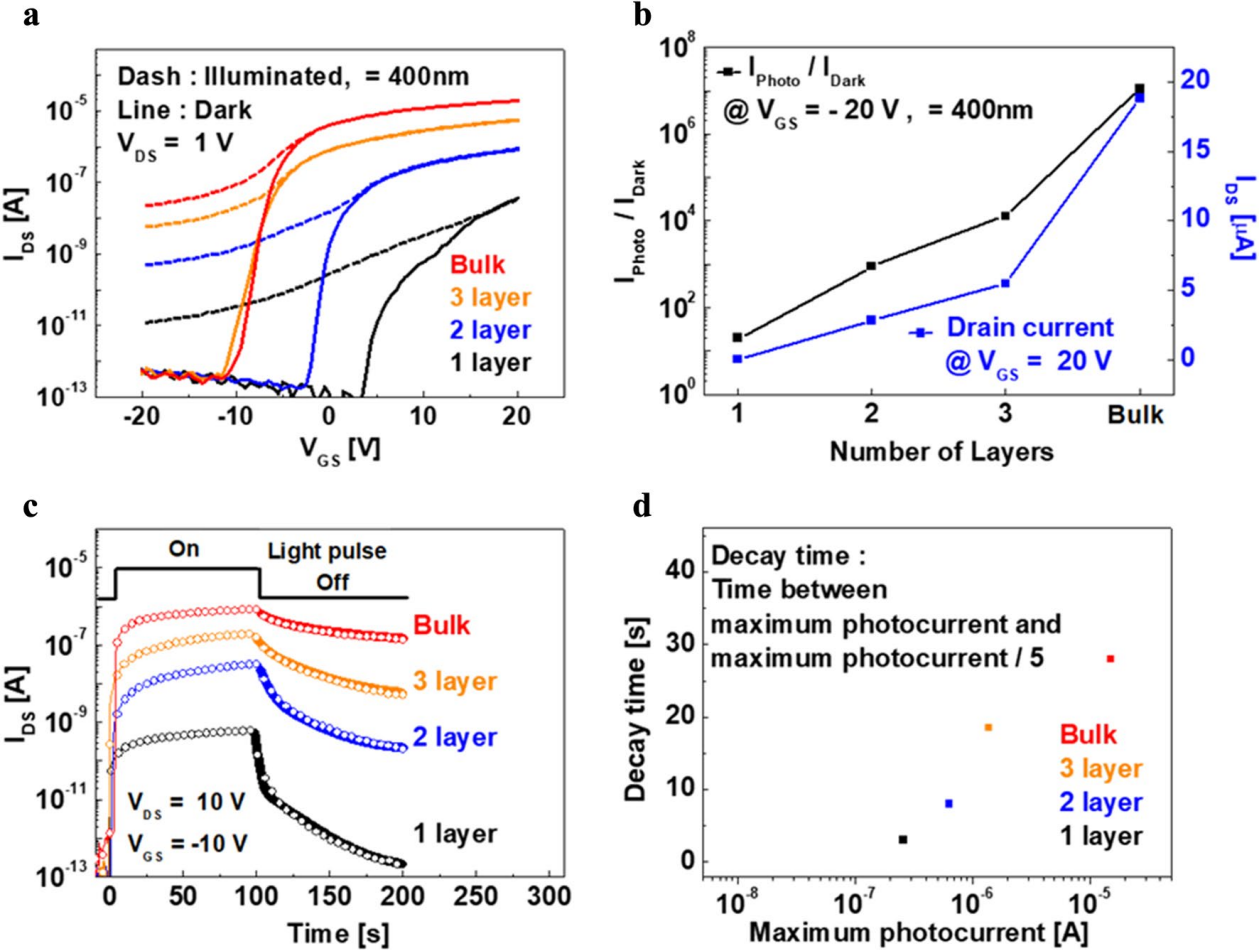
**a** I_DS_–V_GS_ characteristics under dark and illuminated conditions (λ = 400 nm and power ∼ 50 mW/cm^2^), **b** ratio between I_Photo_ and I_Dark_ (left side axis) at V_GS_ = − 20 V and I_DS_ (right side axis) at V_GS_ 20 V, **c** dynamic photosensitivity under pulsed illumination, and **d** decay time and maximum photocurrent (obtained from Fig. 4.2c) of MoS_2_ photo-TFT samples with different numbers of MoS_2_ layers

Figure 2a presents the I_DS_–V_GS_ characteristics of the MoS_2_ phototransistors with different layer thicknesses under dark and illuminated conditions. An increase in the dark-state ON current as well as the photocurrent in the illuminated state was observed with an increase in the MoS_2_ thickness from the monolayer to the bulk. Figure 2b shows the photocurrent (I_Photo_) to dark current (I_Dark_) ratio in the off state and the drain current in the on state as a function of the layer thickness. The increase in the drain current with the layer thickness can be explained by the increased carrier concentration. The effects of the layer thickness on the persistent photoconductivity (PPC) of the fabricated MoS_2_ phototransistors are shown in Fig. 2c. The PPC measurements were carried out by exposing the MoS_2_ TFTs to light pulses at a wavelength of 400 nm with a fixed intensity (5 mW/cm^2^). Figure 2d shows the decay time with the maximum photocurrent for the different layers obtained from Fig. 2c. Here, the decay time represents the time required for the photocurrent to decrease from the maximum level to one-fifth of its maximum value. It can be seen that the decay time and the magnitude of the maximum photocurrent of the phototransistors increase with an increase in the layer thickness.

**Fig. 3:**
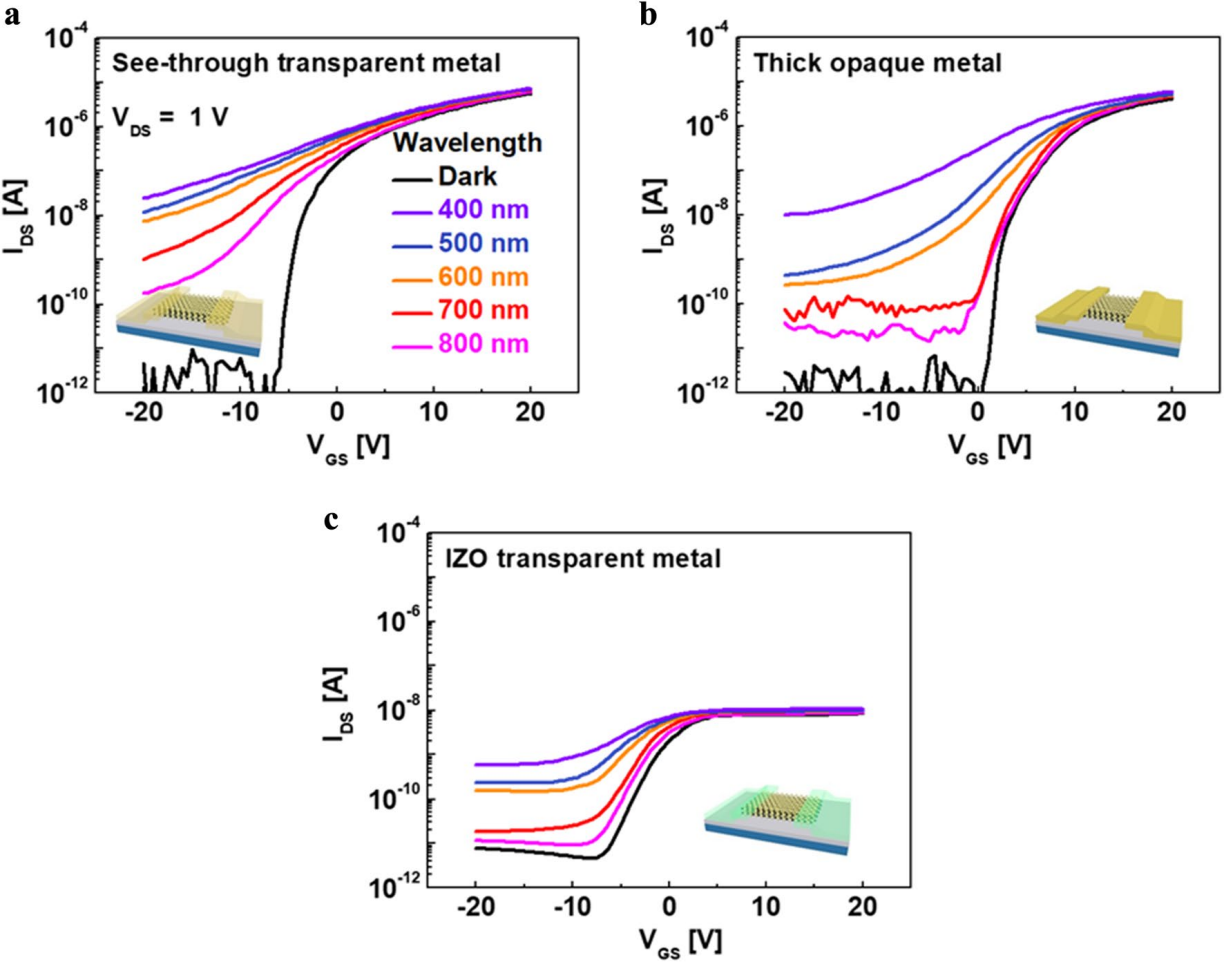
I_DS_–V_GS_ characteristics of the multi-layer MoS_2_ phototransistor with **a** see-through metal, **b** thick opaque metal and **c** IZO transparent metal in the dark and under illumination at different wavelengths

The optical property of the as fabricated MoS_2_ phototransistors were significantly enhanced by adapting see-through transparent electrodes instead of the traditional global bottom gate or patterned local bottom gate structures and were compared with those of conventional opaque electrodes and transparent IZO electrodes. Figure 3a–c show the I_DS_–V_GS_ characteristics of MoS_2_ phototransistors with various metal electrodes. This measurement was carried out under both dark and light conditions using a focused laser with different wavelengths at steps of 100 nm.

From Fig. 3c, it can be seen that the use of IZO transparent metals limits the optical performance of the MoS_2_ phototransistor due to the high Schottky barrier resulting from the Fermi-level pinning effect caused by its high workfunction (~ 5 eV). Furthermore, the sheet resistance of the IZO metal electrode obtained from four-point probe measurements was as high as 105 Ω/square. On the other hand, the sheet resistance of the see-through metal electrode was 8 Ω/square, much lower than that of IZO, which is not significantly different from the value of 1.4 Ω/square, which is the sheet resistance of a conventional Ti/Au metal electrode. The see-through metal was chosen not only for its sheet resistance properties but also for its transmittance capabilities. This see-through metal electrode shows transmittance of 70% under visible light of 532 nm and allows incident light to reach the entire channel area below the source and drain electrodes.

**Fig. 4:**
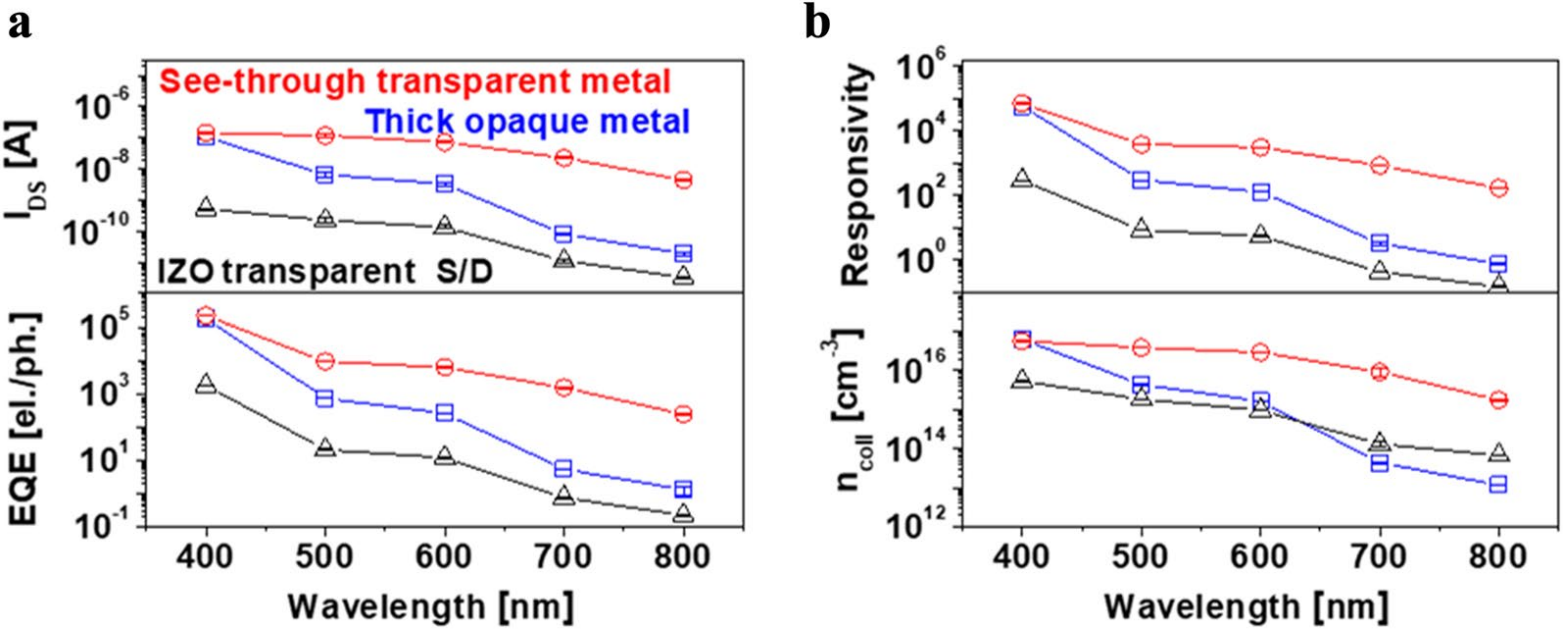
**a** I_Photo_ and external quantum efficiency (EQE), **b** responsivity and collected carrier density values of multi-layer MoS_2_ phototransistors with see-through metal, thick opaque metal and IZO transparent metal electrode under illumination at different wavelengths

The optical properties of the external quantum efficiency (EQE), responsiveness (R) and collected carrier density (n_coll_) of the phototransistors were extracted and calculated from Eqs. (1), (2) and (3) as a function of the wavelength depending on the different contact electrodes [21, 22]. These results are shown in Fig. 4.

1 $$ {\text{EQE}} = \frac{{I_{DS} /q}}{{P_{i} /h\upsilon }} $$

2 $$ {\text{R}} = \frac{{J_{total} - J_{dark} }}{{P_{i} }} $$

3 $$ \eta_{coll} = \frac{{I_{DS} }}{{q\mu_{FE} \left( {W/L} \right)t_{s} V_{DS} }}. $$

Here P_i_ is the power density in the illumination state, hν is the incident photon energy, J_Total_ is the current density in the illumination state, J_Dark_ is the current density in the dark state, μ_FE_ is the field-effect mobility of each device and t_S_ is the MoS_2_ layer thickness.

As shown in Fig. 4, MoS_2_ phototransistors with see-through metal electrodes exhibit significantly improved optical properties as compared to the thick opaque or IZO transparent metal in the visible region. This results from the enhancement in the photocurrent due to the penetration of incident light to the entire active region below the transparent electrode, as described above.

**Fig. 5:**
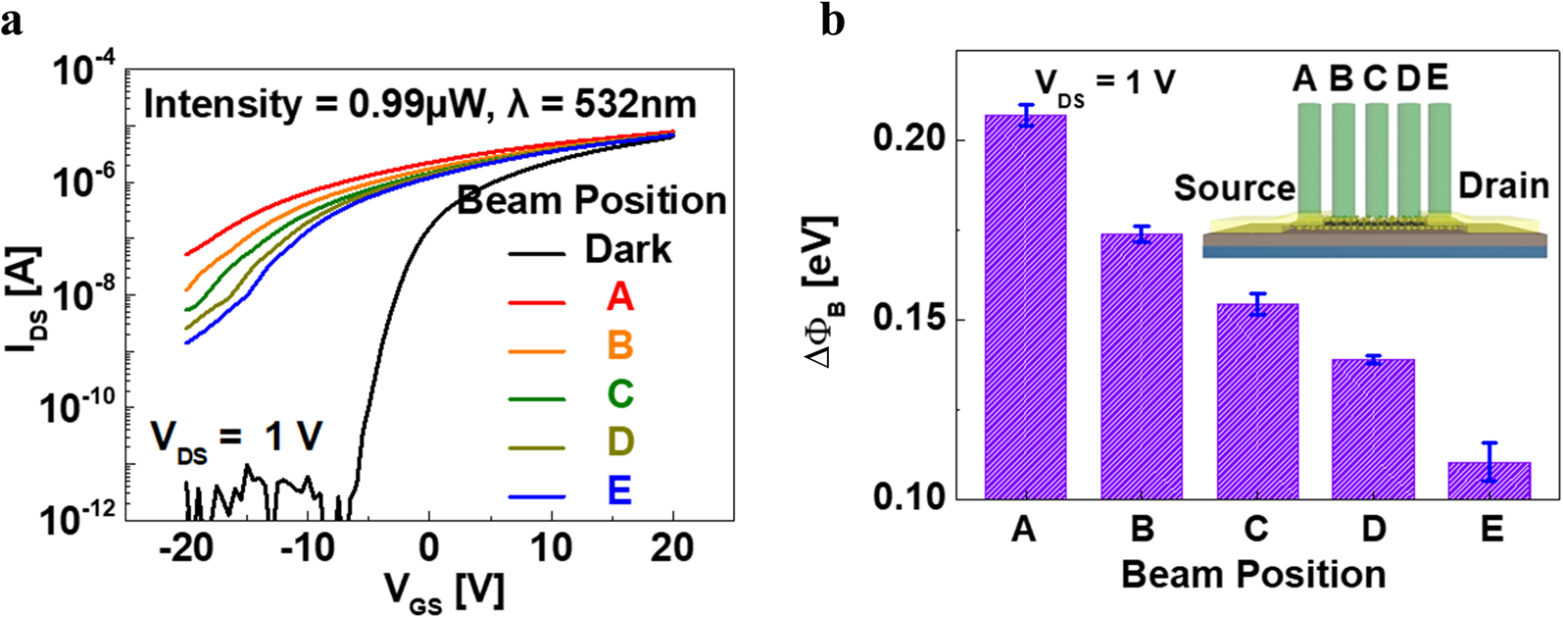
**a** I_DS_–V_GS_ curves and **b** variations in the barrier height with the beam position of the multi-layer MoS_2_ phototransistors (Inset: 3D schematic image of a MoS_2_ phototransistor representing the different beam positions)

To identify the photocurrent mechanism, the photocurrent of the multi-layer MoS_2_ phototransistor with the see-through metal electrode was measured by locally illuminating the MoS_2_ channel at different positions (inset of Fig. 5b). A beam with a wavelength of 532 nm at an intensity level of 0.99 μW was used for this purpose. As indicated by the I_DS_–V_GS_ characteristics presented in Fig. 5b, the photocurrent of the MoS_2_ TFT is highest when the beam is located at the source position (A), after which it decreases along the channel (B, C, D), and is lowest at the drain (E). This can be explained by the barrier height variation (BHV), i.e., Δφ_B_, between the source and the channel due to the incident light. The BHV in this case is mainly caused by the electrostatic force induced at the junction between the metal and the semiconductor. It can be expressed by the following equation.

4 $$ I_{DS} = I_{DS0} \exp \left( {\alpha \frac{{q\Delta \varPhi_{B} }}{kT}} \right). $$

Here I_DS0_ is a reference current value without variation of the barrier height, α is a constant and kT is the thermal energy at room temperature.

Figure 5b shows the BHV depending on the beam position obtained from Eq. (4). As expected, the BHV at the source position has the largest value. Due to the increased BHV, a greater amount of electron injection (n_inj_) occurs from the source and causes an increase in the photocurrent [23]. From Eqs. (3) and (4), n_inj_ can be deduced as follows:

5 $$ I_{DS} = I_{DS0} \exp \left( {\alpha \frac{{q\Delta \varPhi_{B} }}{kT}} \right). $$

Here n_0_ is a constant indicating the reference carrier density.

## Conclusion

In summary, we fabricated mono-layer to multi-layer MoS_2_ TFTs and evaluated their electrical and optical properties. Increases in the dark-state ON current as well as the photocurrent in an illuminated state were observed along with an increase in the decay time (as found in the PPC measurement results) with an increase in the MoS_2_ thickness from the monolayer to the bulk due to the increased carrier concentration. To improve the optical properties, a see-through metal electrode with a very thin film of Ti/Au metal was introduced. This see-through metal electrode showed transmittance of 70% or more under visible light at 532 nm. MoS_2_ phototransistors with see-through metal electrodes exhibit significantly improved optical properties as compared to thick opaque or IZO transparent metal samples in the visible light region. Furthermore, photocurrent measurements with respect to the position of the beam along the MoS_2_ channel revealed that more electrons are injected from the source when the beam is placed on the source side due to the reduced barrier height, giving rise to a significant enhancement in the photocurrent. We hope that the results presented here can provide considerable help to those attempting to understand the photocurrent mechanism as well as the origin of the improved high photocurrent in these types of devices.

## Data Availability

Not applicable.
